# Hepatitis E Virus Infection in Brazil: A Scoping Review of Epidemiological Features

**DOI:** 10.3390/pathogens14090895

**Published:** 2025-09-05

**Authors:** Carolline Araujo Mariz, Lílian Rose Maia Gomes de Araújo, Edmundo Pessoa Lopes

**Affiliations:** 1 Department of Parasitology, Institute Aggeu Magalhães, Oswaldo Cruz Foundation, Recife 50740-465, PE, Brazil; 2 Department of Gastroenterology, Hospital das Clínicas/EBSERH, Federal University of Pernambuco, Recife 50670-901, PE, Brazil; maia_lilian@hotmail.com; 3 Postgraduate Program in Tropical Medicine, Center of Medical Sciences, Federal University of Pernambuco, Recife 50740-600, PE, Brazil

**Keywords:** hepatitis E, serosurvey, prevalence, Brazil, genotype 3, epidemiology, anti-HEV IgG

## Abstract

Introduction: Although Brazil includes industrialized regions, such as the Southeast, it also has underdeveloped areas with poor sanitation, such as the North and Northeast, resembling regions in Africa and Asia where HEV is endemic. In Brazil, HEV is suspected to occur mainly as a zoonosis. Given the wide variation in HEV prevalence across the five regions, a scoping review was conducted to systematically evaluate its prevalence and circulating genotypes. Aim: To investigate the epidemiological characteristics of HEV in Brazil, including modes of transmission, by reviewing genotyping studies in humans and swine. Methods: This scoping review followed the methodological framework of the Joanna Briggs Institute (JBI) and the PRISMA-ScR checklist. Gray literature was retrieved from Google Scholar, the Brazilian Digital Library of Theses and Dissertations, and the Thesis and Dissertation Catalog of the Coordination for the Improvement of Higher Education Personnel. Searches were performed in June and July 2025 in MEDLINE and LILACS. The evidence on HEV epidemiology in Brazil was mapped using the Population, Concept, and Context strategy. Results: Among 57 studies on HEV prevalence in Brazil, 45 (78.9%) involved humans and 12 (21.1%) involved swine. IgG prevalence ranged from 0.5% in the North to 59.4% in the South. IgM prevalence was lowest in the Northeast (0.1%) and highest in the North (16.3%). In swine, HEV was detected in all regions, with variation in sample types, husbandry practices, and prevalence. Genotyping revealed exclusively HEV-3 in all regions where analysis was performed. Conclusions: HEV infection is present throughout Brazil, with higher prevalence in the South and Southeast. The circulating genotype is HEV-3, and transmission is likely linked to swine breeding and consumption.

## 1. Introduction

Hepatitis E virus (HEV) is the leading cause of acute enterically transmitted hepatitis worldwide [1]. According to recent World Health Organization (WHO) estimates, in 2021 there were approximately 20 million cases and 3500 deaths attributable to hepatitis E globally [2,3].

HEV belongs to the family *Hepeviridae*, genus *Orthohepevirus*, species A, and is classified into eight genotypes (HEV-1 to HEV-8) [4]. Among these, genotypes 1 to 4 have been identified in humans, while genotypes 5 to 8 are restricted to animals [5]. Genotypes 1 and 2 are transmitted via the fecal–oral route through contaminated water and are prevalent in regions with inadequate sanitation, particularly in Africa and Asia [6]. Although sporadic cases are frequent, large-scale outbreaks have also been documented, sometimes affecting thousands of individuals [7]. Genotypes 3 and 4, by contrast, are primarily zoonotic, transmitted through the consumption of undercooked meat or direct contact with swine. These infections are usually acute, asymptomatic, and self-limiting [8,9], but in immunocompromised patients, they may progress to chronic hepatitis and even cirrhosis [10].

In recent decades, autochthonous HEV infections have been increasingly reported in industrialized countries in Europe and North America, mainly associated with genotypes 3 and 4. These cases display distinct epidemiological and clinical patterns compared with genotypes 1 and 2, which predominate in developing countries [8,11].

A large meta-analysis assessing the global prevalence of HEV, including 287 studies and 1,099,717 participants, reported an overall anti-HEV IgG prevalence of 12.47%. The data, stratified across 75 countries and six continents, showed the highest seroprevalence in Africa (21.76%), followed by Asia (15.80%), Europe (9.31%), North America (8.05%), South America (7.28%), and Oceania (5.99%). HEV-1 infections were found to occur mainly in India and China, whereas HEV-3 predominated in European countries [12].

In Latin America and the Caribbean, a recent systematic review and meta-analysis estimated the overall prevalence of hepatitis E at 9.0%, with substantial heterogeneity (I^2^ = 97.3%) and values ranging from 0% to 36% [13]. The lowest prevalence was reported in Brazil, while the highest was observed in Cuba [14,15,16].

In Brazil, a systematic review and meta-analysis conducted a few years ago found an overall anti-HEV prevalence of 6% (95% CI: 5.0–7.0), with marked heterogeneity across studies (I^2^ = 86.7%) [17]. Reported prevalence ranged from 0% (95% CI: 0.0–3.0) in Amazonas, in the northern region [18], to 10.0% (95% CI: 7.0–15.0) in Santa Catarina, in the southern region [19], underscoring the wide regional variation in HEV infection [17].

Because of the short duration of HEV viremia and the predominance of cross-sectional study designs, data on circulating genotypes in Brazil remain limited. Nevertheless, the absence of epidemic outbreaks and findings from studies in swine suggest that HEV-3 is the predominant genotype [9]. Although Brazil includes industrialized regions, such as the Southeast, it also encompasses underdeveloped areas with poor sanitation, such as the North and Northeast, resembling regions of Africa and Asia where HEV-1 is common [20]. This raises the possibility that HEV may circulate in Brazil as a zoonotic pathogen.

Given the marked regional variation in HEV prevalence across Brazil, we conducted a scoping review to systematically map the available research and identify knowledge gaps. The central research question guiding this review was: What are the epidemiological characteristics of HEV infection across the regions of Brazil?

## 2. Materials and Methods

This scoping review was conducted following the methodological framework proposed by the Joanna Briggs Institute (JBI) [21] and reported in accordance with the Preferred Reporting Items for Systematic Reviews and Meta-Analyses extension for Scoping Reviews (PRISMA-ScR) checklist [22].

### 2.1. Eligility Criteria

The eligibility criteria for this review were defined as follows: publications addressing the epidemiology of HEV infection in Brazil from 1995 onwards, when the first reports of the disease were documented in the country, with no language restrictions. Eligible studies included primary and secondary, empirical, quantitative research, with preference given to cohort, case-control, and cross-sectional designs. Excluded were letters to the editor, validation studies, review articles, case reports, conference abstracts, incomplete articles, studies in the project phase, and studies lacking results. Articles focusing on HEV epidemiology outside Brazil and those addressing contamination in mollusks or other animals (e.g., horses, capybaras, wild boars) were also excluded.

### 2.2. Information Sources

Searches were carried out in June and July 2025 in the following databases: Medical Literature Analysis and Retrieval System Online (MEDLINE) via PubMed, MEDLINE via the Virtual Health Library (VHL), and Latin American and Caribbean Health Sciences Literature (LILACS). Gray literature was retrieved from Google Scholar, the Brazilian Digital Library of Theses and Dissertations (BDTD), and the Thesis and Dissertation Catalog (CTD) of the Coordination for the Improvement of Higher Education Personnel (CAPES).

### 2.3. Search Strategy

A search strategy was developed to identify evidence on the epidemiology of HEV infection in Brazil. The complete search equation is presented in Table 1.

### 2.4. Selection of Sources of Evidence

Results retrieved from the databases were exported to Microsoft Excel^®^ for independent screening by two reviewers, with discrepancies resolved by a third reviewer. In the first phase, titles and abstracts were screened; in the second phase, full-text articles meeting the inclusion criteria were assessed. Additionally, the reference lists of included studies were manually reviewed to identify further eligible publications.

### 2.5. Data Charting Process and Data Items

Data extraction was performed independently by two reviewers using Microsoft^®^ Excel^®^ for Microsoft 365 MSO. The extracted information was verified by a third reviewer, with disagreements resolved through discussion until consensus was reached. Data charting followed the JBI tool for study characterization [21]. The extraction table included: authorship, journal of publication, country of origin, year of publication, objectives, study design, sample size, and main results regarding HEV seroprevalence in the five regions of Brazil.

## 3. Results

**Figure 1:**
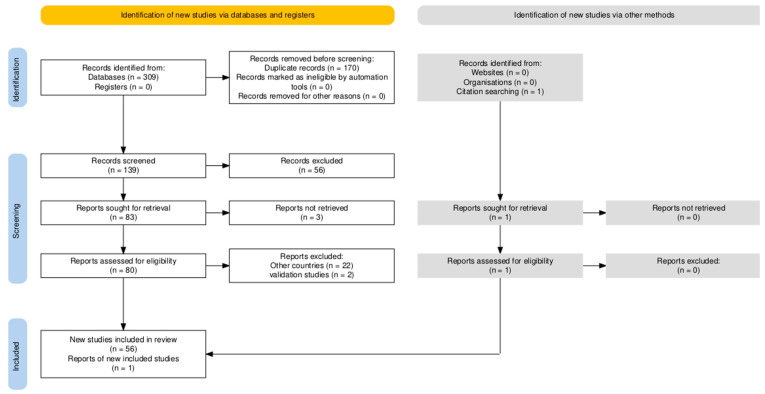
PRISMA Flow Diagram, generated with the PRISMA tool [23], which schematically illustrates the article selection process.

Initially, 309 publications were identified in the MEDLINE and LILACS databases, and one additional record was retrieved through citation searching (Figure 1).

After removing 170 duplicates, a total of 140 references were screened by reading their abstracts. Of these, 83 were excluded, resulting in 57 studies selected for full-text assessment, all of which were included in the final review. Among the 57 studies on the prevalence of hepatitis E virus infection in Brazil, 45 (78.9%) investigated humans and 12 (21.1%) focused on swine.

The distribution of the 45 studies involving HEV in humans is shown in Table 2. Most were conducted in the Southeast region (35.5%), predominantly in the state of São Paulo (87.5%), followed by the Northeast (20%) and Central-West (20%). The North region accounted for the fewest studies (11.1%), of which three (60%) were in Pará, one (20%) in Acre, and the remaining two (20%) in Amazonas and Rondônia.

**Figure 2:**
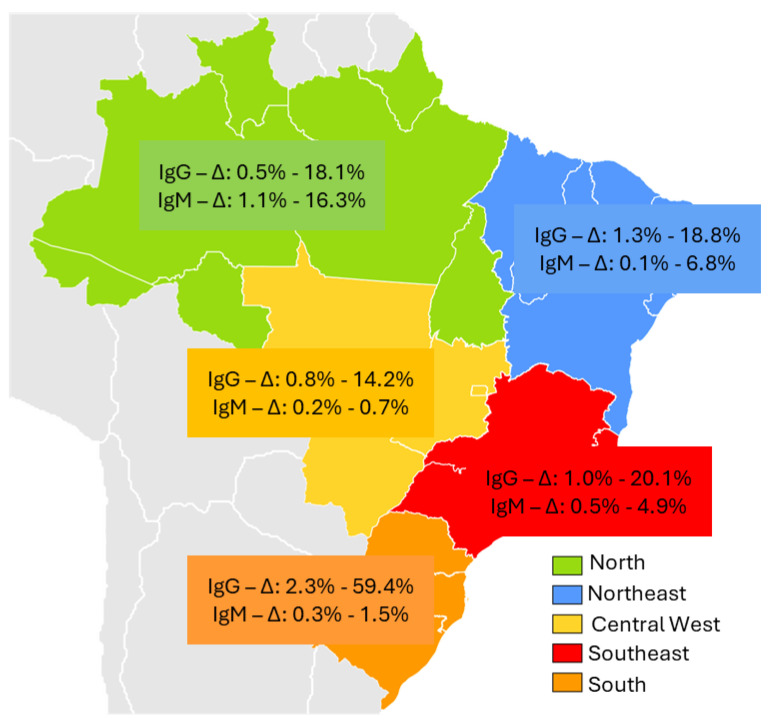
Prevalence of HEV infection markers (IgG and IgM) in the Brazilian population by region, 1995–2025. Gray-shaded areas on the map correspond to South American countries bordering Brazil; IgG-∆ and IgM-∆: represents the delta of IgM and IgG immunoglobulin variation reported in the study population.

Regional variations in HEV prevalence are presented in Figure 2. The overall prevalence of the IgG marker in Brazil ranged from 0.5% in the North to 59.4% in the South. Conversely, the prevalence of the IgM marker was lowest in the Northeast (0.1%) and highest in the North (16.3%).

Among the 12 studies involving swine, HEV infection was detected in all regions of Brazil, with variation in sample types, husbandry practices, and prevalence rates. These findings are summarized in Table 3.

**Figure 3:**
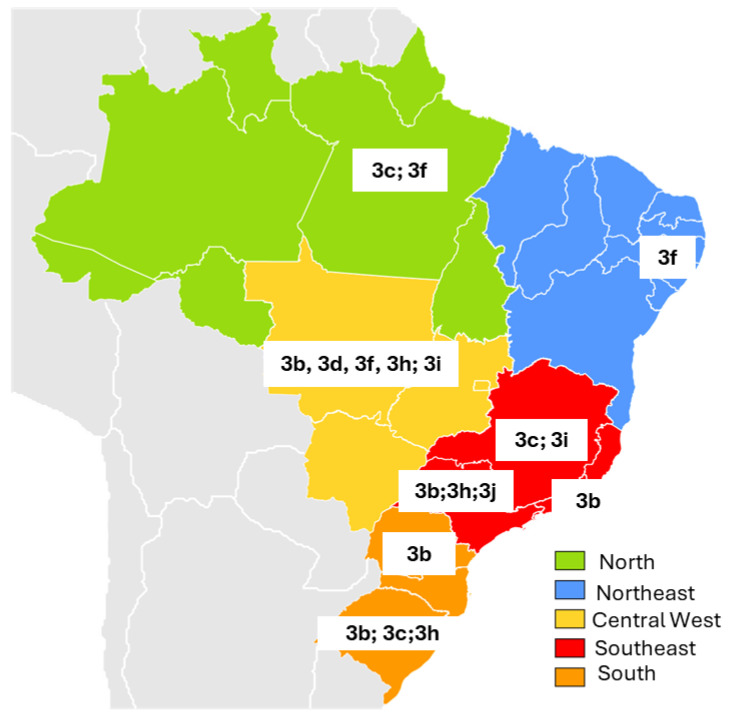
Distribution of HEV genotype 3 phylogenetic subtypes across the five major regions of Brazil, 1995–2025. Gray-shaded areas on the map correspond to South American countries bordering Brazil; 3b, 3c, 3d, 3h, 3i, 3j and 3f: refer to HEV genotype 3 subtypes.

Across all regions where genotypic analysis was performed, only HEV genotype 3 (HEV-3) was identified. Intra-genotypic diversity was demonstrated by the distribution of distinct phylogenetic subtypes across states, as illustrated in Figure 3.

In the North Region, specifically in the state of Pará, HEV subtypes 3c and 3f were identified. In the Northeast, isolates from the state of Pernambuco were classified as subtype 3f. In the Central-West Region, in Mato Grosso, multiple subtypes were reported: 3b and 3f in one study, and 3d, 3h, and 3i in another, highlighting the genetic diversity of HEV in this region. In the Southeast, subtype 3b was detected in Rio de Janeiro, while in Minas Gerais the viruses were classified as subtypes 3c and 3i. In São Paulo, subtypes 3b, 3h, and 3j were reported. In the South, subtype 3b was identified in Paraná, whereas in Rio Grande do Sul the circulating subtypes included 3b, 3c, and 3h.

## 4. Discussion

Recent estimates for hepatitis B and C viruses suggest a declining trend in the incidence and prevalence of these infections in Brazil, likely as a result of vaccination and the availability of antiviral therapy in recent years [79,80]. In contrast, data on the occurrence of HEV infection remain scarce, possibly due to the limited availability of anti-HEV testing in the Public Unified Health System. Currently, such tests can only be performed in reference laboratories (e.g., LAHEP/Fiocruz) upon institutional referral [81].

Brazil is a country of vast territorial extension and a highly diverse population shaped by extensive ethnic and cultural admixture. Cultural influences from Indigenous peoples persist mainly in the North and Central-West regions, Portuguese heritage is predominant in the Northeast, while Italian and German influences are more evident in the Southeast and South [82]. Furthermore, African cultural contributions, introduced during the 17th to 19th centuries, are present throughout the country. This complex historical background likely contributes to the wide variability in the prevalence and epidemiological characteristics of HEV across Brazil [82].

The heterogeneity of studies conducted in Brazil, reflected in approximately 48 publications over the past 30 years, poses challenges for obtaining robust, nationwide data. These studies evaluated diverse population groups across the five major regions of the country and employed various laboratory tests (Table 2). Nevertheless, a general analysis of the data indicates a trend of increasing HEV prevalence from the North to the South, with intermediate rates observed in the Central-West and Northeast, as reported by several authors [9,83]. Socio-demographic factors, such as higher education levels, greater purchasing power, and advanced industrialization in the Southeast and South regions, may partly explain this pattern [84].

Epidemiological data further reveal pronounced regional variation, with higher HEV occurrence in the Southeast and South regions. Some authors have suggested that these differences could be influenced by variability in the sensitivity of ELISA assays from different manufacturers [19,85]. However, more recent studies using updated testing methodologies indicate that such differences in anti-HEV IgG detection may be less significant than previously thought [12,54,83].

Studies assessing HEV prevalence in the general Brazilian population are limited, with most investigations focusing on blood donors or specific high-risk groups. Early studies primarily evaluated anti-HEV occurrence in patients with acute hepatitis of undetermined etiology (non-A, non-C hepatitis), in addition to blood donors, and relied on older ELISA assays. More recent investigations have targeted high-risk populations, including intravenous drug users, hemodialysis patients, HIV-positive individuals, transplant recipients, and patients with underlying chronic liver diseases, as summarized in Table 2 [24,25,26,27,28,29,30,31,32,33,34,35,36,37,38,39,40,41,42,43,44,45,46,47,48,49,50,51,52,53,54,55,56,57,58,59,60,61,62,63,64,65,66].

One of the first rigorously designed epidemiological studies in Brazil was conducted in São Paulo, employing active household-based sampling across all neighborhoods and social strata. Among 1059 individuals evaluated, the prevalence of anti-HEV IgG was estimated at 1.68%, with higher rates observed in older individuals and residents of the West and downtown areas [46]. Shortly thereafter, a similar study in the Manguinhos community of Rio de Janeiro (Southeast region) reported a prevalence of 2.4% among 699 participants [61]. More recently, in a small municipality of São Paulo state, anti-HEV IgG prevalence reached 20% among 248 individuals, with a significant association between seropositivity and the consumption of raw meat [54]. In this region, the local spread of HEV may have been facilitated by wild boars that interbred with domestic pigs, contributing to viral transmission [54].

Among blood donors, HEV prevalence appears to increase in the South region of Brazil. The first study published in the country, in 1997, evaluated 200 blood donors from Salvador (Northeast region) and reported an anti-HEV prevalence of 2% [15]. Subsequent investigations in the same region found prevalences of 0.9% among 996 donors in Recife and 1.35% among 890 donors in Teresina [33,36]. In contrast, studies in the South region showed higher rates, with 10% prevalence among 300 donors in Santa Catarina [19], and 7.1% and 18.7% among 281 and 80 donors, respectively, in Rio Grande do Sul [64,65].

Across nearly all studies, anti-HEV prevalence increases with age, a trend also described in European meta-analyses and likely reflecting cumulative exposure over time [43,50,53,86]. Some studies further suggest that longer exposure to risk factors, such as extended crack cocaine use, prolonged HIV infection, or extended residence in rural settlements, is associated with higher anti-HEV prevalence [28,32,39].

Additionally, Brazilian studies indicate higher anti-HEV prevalence among patients with advanced liver disease. Several reports documented elevated rates in cirrhotic patients with advanced fibrosis and in individuals with diabetes mellitus [57,65]. Two studies conducted in the Brazilian Northeast, where Schistosomiasis mansoni is endemic, found high anti-HEV prevalence in patients with this parasitic infection. Moreover, HEV markers were associated with more severe forms of schistosomiasis, suggesting that the virus may exacerbate disease progression or that patients with advanced parasitic disease are at greater risk of HEV exposure [31,35].

The elevated risk of HEV infection in patients with schistosomiasis may be attributable to insufficient sanitation and limited access to treated water in endemic areas. Several studies in Brazil have demonstrated that regions with inadequate sewage systems, such as rural settlements, are associated with increased HEV exposure [39,42,45].

These observations suggest that HEV genotypes 1 and 2, which are transmitted via the fecal-oral route as in Africa and Asia, could circulate in Brazil [7,8]. However, in areas lacking proper sanitation, rural practices such as domestic swine farming and consumption of game meat are common, favoring zoonotic transmission linked to genotypes 3 and 4 [7,8,54,68].

Brazilian studies assessing HEV prevalence in swine report high infection rates in four regions of the country (except the North), ranging from 60% to 80%. Genotyping consistently identified HEV-3 in almost all studies (Table 3). Furthermore, in the four human studies that performed genotype analysis, HEV-3 variants were detected in all cases [28,49,58,64]. Sequencing of HEV-RNA isolated from humans in some studies closely matched the HEV sequences from swine in the same regions, strongly suggesting zoonotic transmission [64,77].

Notably, in the Southeast and South regions of Brazil, which were historically influenced by Italian and German cultures, the temperate climate favors the domestic raising of swine for meat and smoked products during winter. These farms are often small-scale and inadequately regulated by health authorities. Additionally, in some interior regions of Brazil, the consumption of beef offal and wild animal meat is common. HEV genotypes isolated from these animals were consistently identified as HEV-3 [87,88].

Despite the strong evidence of the circulation of HEV-3 in Brazil, we unexpectedly identified two articles in the literature review that suggest this infection is endemic and present maps indicating the occurrence of HEV-1 in this country. However, in these two articles, it is unclear where the information regarding the endemicity and circulation of HEV-1 in Brazil originated [20,89]. Additionally, there are reviews that indicate the HEV circulating in Brazil is genotype 3 [12,90,91].

The primary limitation of this review is the scarcity of robust studies involving large, representative samples across all five regions of Brazil, including both major cities in industrialized areas and small rural municipalities. Furthermore, heterogeneity in study designs, sample sizes, ELISA kits employed, and the characteristics of evaluated populations presents additional challenges.

In conclusion, the evidence indicates that HEV infection is present throughout all five regions of Brazil, with higher prevalence in the South and Southeast. The circulating genotype is predominantly HEV-3, and transmission is likely associated with swine breeding and consumption.

## Figures and Tables

**Table 1:** Application of the population, concept, and context (PCC) strategy in the scoping review.

**Objective/** **Problem**	To investigate the epidemiological characteristics of HEV in Brazil, including modes of transmission, by reviewing genotyping studies in humans and swine/What are the epidemiological characteristics of HEV infection across the regions of Brazil?		
	**P**	**C**	C*
**Extraction**	Epidemiology	Hepatitis E	Brazil
**Combination**	epidemiology; epidemiologia	hepatitis E; hepatitis E virus; Hepatite E; vírus da hepatite E	brazil; brasil
**Construction**	(“epidemiology” **OR** “epidemiologia”)	(“hepatitis E” **OR** “hepatitis E vírus” **OR** “hepatite E” **OR** “vírus da hepatite E”)	(“Brazil” **OR** “Brasil”)
**Use**	(“epidemiology” **OR** “epidemiologia”) **AND** (“hepatitis E” **OR** “hepatitis E vírus” **OR** “hepatite E” **OR** “vírus da hepatite E”) **AND** (“Brazil” **OR** “Brasil”); (“hepatitis E” **OR** “hepatitis E vírus”) **AND** (“Brazil” **OR** “Brasil”); (“hepatitis E/epidemiology” **OR** “hepatitis E vírus”) **AND** (“Brazil”)		

P = Population; C = Concept; C* = Context.

**Table 2:** Articles published between 1995 and 2025 reporting the prevalence and epidemiological characteristics of HEV markers in humans in Brazil, stratified by the five major regions of the country.

Brazil Region	Type of Study	Selected Population	Epidemiological Characteristics	Sample Size	Anti-HEV Prevalence		RNA	Genotype	Author/Year
					IgG n (%)	IgM n (%)			
**North**									
**Acre**	Retrospective cross-sectional	Residents of an agricultural settlement in 2004	Age > 21 years	388	50 12.8%	7 16.3%	n/a	n/a	Vitral CL et al., 2014 [24]
**Amazônia/ Rondônia**	Cross-sectional	Yanomani Indians Urban and rural areas	HEV in urban areas (2.9%), rural areas (14.2%) and village areas (2.8%)	811	55 6.8%	n/a	n/a	n/a	Vasconcelos MP et al., 2024 [25]
**Pará**	Cross-sectional	Afro-descendant community	Young men reported eating bushmeat	535	3 0.5%	6 1.1%	negative	n/a	Souza AJS et al., 2018 [26]
	Cross-sectional	Suspected cases of acute hepatitis	Male gender (55.2%)	318	29 9.1%	16 5.0%	negative	n/a	Souza AJS et al., 2019 [27]
	Cross-sectional	Crack cocaine users	Poorer and homeless; longer use of crack cocaine	437	79 18.1%	6 1.4%	positive	3c	Nascimento RS et al., 2021 [28]
**Northeast**									
**Bahia**	Retrospective cross-sectional	Blood donors (n = 200) Hemodialyzed (n = 392)	Blood donors	200	4 2%	n/a	n/a	n/a	Paraná R et al., 1997 [15]
	Cross-sectional	Acute sporadic non-A, non-B (NANB)	Aminotransferases elevation	43	5 12%	negative	n/a	n/a	Paraná R et al., 1999 [29]
	Cross-sectional	Patients with acute viral hepatitis	Higher prevalence of HEV in patients with acute hepatitis	73	21 28.8%	5 6.8%	n/a	n/a	Lyra AC et al., 2005 [30]
**Pernambuco**	Retrospective cross-sectional	Patients with schistosomiasis mansoni	Patients treated at a referral hospital with advanced forms of the disease	80	15 18.8%	negative	negative	n/a	Passos -Castilho AM et al., 2016 [31]
	Cross-sectional	People living with HIV/AIDS	Higher HIV infection time	366	15 4.1%	n/a	negative	n/a	Bezerra LA et al., 2019 [32]
	Cross-sectional	Blood candidates and donors	All male gender, consumption of pork and chicken	996	9 0.9%	n/a	n/a	n/a	Cunha GG et al., 2022 [33]
	Cross-sectional	Patients with chronic liver disease	Contact with swine and more advanced liver disease	227	7 3.1%	n/a	negative	n/a	Araújo LRMG et al., 2024 [34]
	Retrospective cross-sectional	Patients with schistosomiasis mansoni	More advanced periportal fibrosis	286	15 5.2%	negative	negative	n/a	Gomes CTO et al., 2024 [35]
**Piaui**	Cross-sectional	Blood donors	Male gender (66.7%), age ≥ 30 years (75%)	890	12 1.3%	1 0.1%	negative	n/a	Silva-Sampaio JP et al., 2025 [36]
**Central West**									
**Goiás**	Prevalence survey	Recyclable material collectors	Contact with human feces (87.5%) and animal feces (75%)	431	22 5.1%	3 0.7%	negative	n/a	Martins RM et al., 2014 [37]
	Cross-sectional	Patients with acute viral hepatitis	Pork consumption (95%) and wild animal (75%)	379	20 5.3%	1 0.3%	negative	n/a	Freitas NR et al., 2016 [38]
	Cross-sectional	Rural settlement	Male gender (75%), Time in rural settlement >5 years	464	16 3.4%	n/a	negative	n/a	Freitas NR et al., 2017 [39]
	Cohort	Kidney transplant recipients	Previous hemodialysis (100%); Consumption of wild animal (87.5%)	316	8 2.5%	1 0.3%	negative	n/a	Oliveira JMNS et al., 2018 [40]
	Cross-sectional	Recyclers, immigrants, refugees, and homeless people	Homeless; recyclers	459	4 0.87%	1 0.2%	negative		Teles AS et al. 2023 [41]
**Mato Grosso**	Prevalence survey	School children	Absence of sanitary sewage.	487	22 4.5%	n/a	n/a	n/a	Assis SB et al., 2002 [42]
	Cross-sectional	Swine handlers	age ≥ 50 years, longer exposure to swine	310	26 8.4%	n/a	n/a	n/a	Silva SM et al., 2022 [43]
**Mato Grosso do Sul**	Cross-sectional	Crack users	Low education level (73.7%), unprotected sexual intercourse	698	99 14.2%	2 0.3%	negative	n/a	Castro VOL et al., 2018 [44]
	Retrospective cross-sectional	Blood donors	Male (75%), age ≥ 30 years (70%): lack of sewage system	250	16 6.4%	Negative	n/a	n/a	Weis-Torres SMDS et al., 2022 [45]
**Southeast**									
**São Paulo**	Prevalence survey	General population	n/a	1059	1.68%	n/a	n/a	n/a	Focaccia R et al.,1998 [46]
	Cross-sectional	Blood donors and staff at a university hospital,	Blood donors with elevated ALT, and cleaning staff	375	18 4.8%	n/a	n/a	n/a	Gonçales NS et al., 2000 [47]
	Cross-sectional	Kidney transplant	Transplant patients with elevated aminotransferases	192	28 15%	n/a	20 10%	n/a	Hering T et al., 2014 [48]
	Retrospective cross-sectional	Patients with clinical suspicion of HEV	age ≥ 40 years	2.271	47 2.1%	27 4.9%	1	3b	Passos-Castilho AM et al., 2015 [49]
	Cross-sectional	Blood donors	age ≥ 45 years	500	49 9.8%	1	negative	n/a	Passos -Castilho AM et al., 2017 [50]
	Cross-sectional	Chronic HCV patients	Contact with swines and consumption of pork	618	63 10.2%	negative	n/a	n/a	Bricks G et al., 2018 [51]
	Cross-sectional	People living with HIV	Age ≥ 40 years	354	38 10.7%	5 1.4%	negative	n/a	Ferreira AC et al., 2018 [52]
	Cross-sectional	Chronic HCV patients	Age ≥ 60 years; contact with swine	618	63 10.2%	negative	n/a	n/a	Bricks G et al., 2019 [53]
	Cross-sectional	Residents of a small municipality in São Paulo	Consumption of raw meat	248	50 20.7%	negative	n/a	n/a	Araújo DCA et al., 2020 [54]
	Cross-sectional	Patients in the Emergency Room with altered levels of ALT	Altered levels of ALT	401	n/a	2 of 90 2.2%	16 of 311 5.1%	n/a	Conte DD et al., 2021 [55]
	Cohort	Liver transplants	HBV/HCV coinfected	294	24 8.2%	6 2%	17 5.8%	n/a	Moraes ACP et al., 2021 [56]
	Cross-sectional	Chronic HCV patients	More advanced liver disease; more Type 2 DM,	181	22 12%	3 1.6%	9 4.9%	n/a	Zitelli PMY et al., 2021 [57]
	Cross-sectional	Patients with acute viral hepatitis	Elevated aminotransferases	91	12 13.2%	4 4.4%	1	3f	Ribeiro LB et al., 2024 [58]
	Prospective	Liver transplanted and donors	n/a	190	19 10%	1 0.5%	negative	n/a	Zicker M et al., 2024 [59]
**Rio de Janeiro**	Retrospective cross-sectional	Acute viral hepatitis; hemodialysis; intravenous drug users; blood donors;	n/a	1115	Acute viral hepatitis (2.1%) hemodialysis (6.2%); UDIVs (11.8%); blood donors (4.3%)	n/a	n/a	n/a	Trinta KS et al., 2001 [60]
	Cross-sectional	Manguinhos Community	Age ≥ 40 years	699	17 2.4%	n/a	n/a	n/a	Santos DC et al., 2002 [61]
**South**									
**Paraná**	Cross-sectional	Blood donors	There was no association with sociodemographic variables	996	23 2.3%	n/a	n/a	n/a	Bortoliero AL et al., 2006 [62]
	Cross-sectional	pregnant women (n = 209); female blood donor (n = 199)	Age ≥ 40 years; >3 number of pregnancies	408	91 22.5%	n/a	negative	n/a	Hardtke S et al., 2018 [63]
**Santa Catarina**	Cross-sectional	Blood donors		300	30 10%	1 0.3%	negative	n/a	Passos-Castilho AM et al., 2016 [19]
**Rio Grande do Sul**	Cross-sectional	PLWHA Blood donors	Age ≥ 40 years; poor sanitation; alcohol use	601	42 6.9%	n/a	8 1.3%	3	Moss da Silva SC et al., 2019 [64]
	Cross-sectional	Cirrhosis; crack users; liver transplanted; blood donors	Higher in cirrhosis; crack users; liver transplanted patients and blood donors	400	78 19.5%	6 1.5%	negative	n/a	Costa et al., 2021 [65]
	Cross-sectional	Blood samples were from laboratories	Age ≥ 40 years	3.000	1.783 59.4%	n/a	negative	n/a	Zorzeto R et al., 2021 [66]

IgM: immunoglobulin M; IgG: immunoglobulin G; n/a: not available; HEV: hepatitis E virus; 3c: HEV genotype 3, subtype c; HIV/AIDS: human immunodeficiency virus/acquired immunodeficiency syndrome; ALT: alanine aminotransferase; HCV: hepatitis C virus; HBV: hepatitis B virus; Type 2 DM: Type 2 diabetes mellitus; PLWHA: people living with HIV/AIDS.

**Table 3:** Articles published between 1995 and 2025 reporting the prevalence and genotypic characteristics of HEV markers in swine in Brazil, stratified by the five major regions of the country.

Brazil Region	State	Herd Characteristics	Biological Sample Tested	Total (n=)	Prevalence HEV		RNA	Genotype	Author/Year
					IgG n (%)	IgM n (%)		Subtype	
**North**	Pará	Six-month-old swine from a licensed slaughterhouse (60%) and a slaughterhouse not registered with health regulatory agencies (40%). Samples collected during slaughter.	Serum, feces and liver	151	13 8.6%	0	15 * 9.9%	3c; 3f	Souza AJ et al., 2012 [67]
**Northeast**	Pernambuco	Coming from a slaughterhouse located in the metropolitan region of Recife (30%) and farms in the rural region of the state (70%)	Serum	325	266 82%	-	n/a	n/a	Oliveira-Filho EF et al., 2017 [68]
	Pernambuco	Animals aged two to six months, from farms that use intensive and extensive production systems.	Feces	119	-	-	2 (1.68%)	3f	Oliveira-Filho EF et al., 2019 [69]
**Central West**	Mato Grosso	Four-month-old animals from large-scale farms (50%) and family farms (50%). Overall, 18 (72%) of the 25 swine presented microscopic liver lesions, characterized by fibrosis and portal inflammation.	Bile, liver and feces	25	-	-	15 ** 83.3%	3b; 3f	Costa Lana et al., 2014 [70]
	Mato Grosso	Growing piglets of both sexes, between three and four months of age, and breeding females, between eight and twenty-four months of age, from subsistence farms.	Serum and feces	150	-	-	12 8%	3d; 3h; 3i	Campos CG et al., 2018 [71]
**Southeast**	Rio de Janeiro	Swine ranging in age from 1 to >25 week in four commercial herds	Serum	357	227 63.6%		n/a	n/a	Vitral CL et al., 2005 [72]
	Rio de Janeiro	Healthy animals aged > five months, from three legal slaughterhouses.	Bile	115			11 *** 9.6%	3b	dos Santos DR et al., 2011 [73]
	Minas Gerais	Healthy animals for slaughter at a state slaughterhouse. No macroscopic lesions were observed in the livers of slaughtered swine during bile collection.	Bile	335	-	-	51 15.2%	3c; 3i	Amorim AR et al., 2018 [74]
	São Paulo	Samples from a state swine biobank.	Feces	89	-	-	7 7.86%	3b; 3h; 3j	Cortez A et al., 2021 [75]
**South**	Paraná	Samples came from maturation cycle farms (58.3%) and grow-to-slaughter farms (41.7%). All swine were asymptomatic.	Feces	170	-	-	26 15.3%	3b	Gardinali NR et al., 2012 [76]
	Paraná	Animals aged between four and 16 weeks old from a small rural property in the region.	Feces	170	-	-	34 20%	3b	Passos-Castilho AM et al., 2017 [77]
	Rio Grande do Sul	Animals from farms located near peri-urban areas or landfills, indigenous reservations, and farms that feed swine with food scraps. Samples from two different periods were analyzed: 2012 (50.6%) and 2014 (49.4%)	Serum	1444	1034 71.6%	-	6 **** 0.8%	3b; 3c; 3h	da Silva MS et al., 2018 [78]

* Authors report that, interestingly, in the present study, HEV RNA was detected more frequently among swine without serological evidence of HEV infection: among fifteen swine with positive PCR, only one had detectable anti-HEV IgG. The samples analyzed in the present study were obtained from swine at slaughter age (approximately six months), which may have led to the failure to detect IgM antibodies. ** Among the 18 animals with microscopic liver lesions, HEV RNA was detected in eight (32%) of the swine by nested PCR and in seven (28%) of the swine by IHC in at least one of the samples analyzed from each animal. *** Viral loads observed for bile samples ranged from 101 to 105 genome copies/mL. **** 6/713 samples analyzed for the year 2014. IgM: immunoglobulin M; IgG: immunoglobulin G; n/a: not available; HEV: hepatitis E virus; 3b, 3c, 3d, 3h, 3i, 3j and 3f: refer to HEV genotype 3 subtypes.

## Data Availability

The original contributions presented in this study are included in the article. Further inquiries can be directed to the corresponding author.

## Abbreviations

The following abbreviations are used in this manuscript:

ELISA	Enzyme-Linked Immunosorbent Assay
HBV	Hepatitis B virus
HCV	Hepatitis C virus
HEV	Hepatitis E virus
JBI	Joanna Briggs Institute
PCC	Population, Concept, and Context
PRISMA-ScR	Preferred Reporting Items for Systematic reviews and Meta-Analyses extension for Scoping Reviews
WHO	World Health Organization
